# Maternal oxytocin administration mitigates nociceptive, social, and epigenetic impairments in adolescent offspring exposed to perinatal trauma

**DOI:** 10.1016/j.neurot.2025.e00598

**Published:** 2025-04-22

**Authors:** Sydney Harris, Zoe Kodila, Sabrina Salberg, Marissa Sgro, Elaina Vlassopoulos, Crystal N. Li, Madeleine J. Smith, Sandy R. Shultz, Glenn R. Yamakawa, Melanie Noel, Richelle Mychasiuk

**Affiliations:** a Department of Neuroscience, School of Translational Medicine, Monash University, Melbourne, Victoria, Australia; b Centre for Trauma and Mental Health Research, Vancouver Island University, Nanaimo, B.C., Canada; c Department of Psychology, Alberta Children's Hospital, Hotchkiss Brain Institute, University of Calgary, AB, Canada

**Keywords:** Adverse childhood experiences, Gene expression, Chronic pain, Intergenerational

## Abstract

Adverse childhood experiences (ACEs) alter brain development, leading to vulnerability for chronic pain, mental health disorders, and suicidality. These effects often emerge during adolescence. Importantly, ACEs can occur prenatally, including when exposed to *in utero* intimate partner violence (IPV) or postnatally as maternal neglect. Maternal social support has demonstrated promise in the mitigation of ACE-related deficits. Oxytocin, which has a role in social-bonding and stress regulation, serves as a suitable surrogate for social support in preclinical studies. Therefore, we aimed to explore the effects of oxytocin on alleviating social deficits, nociception, and epigenetic changes resulting from models that aimed to mimic the stress normally induced following exposure to two ACEs: IPV *in utero* and maternal neglect. During pregnancy, dams were randomly assigned to experience the model of IPV or a sham insult. Following birth, offspring from the IPV group underwent 10 days of maternal separation. Dams received three days of oxytocin therapy while nursing. In adolescence, half of the offspring underwent a plantar surgery to induce pain. Overall, in adolescence, rats exposed to the ACEs exhibited increased nociceptive sensitivity and aberrant social interactions, particularly among males, further suggesting that ACEs can increase an individual's risk for chronic pain. The ACEs changed gene expression related to social behaviour and neuroplasticity. Maternal oxytocin normalized pain, social, and gene changes, while oxytocin levels in offspring correlated with nociceptive sensitivity. Although ACEs have enduring consequences, the outcomes are modifiable, and oxytocin may be a robust and implementable therapeutic capable of attenuating early adversity.

## Introduction

In the late 1990's Felliti et al. discovered that early life experiences, particularly adverse childhood experiences (ACEs) could significantly modify physiological health in adulthood [1]. The recognition that ACEs could manifest as increased risk for obesity, cardiovascular disease, and substance abuse disorders changed the health landscape [2,3]. It did not take long for researchers to demonstrate that ACEs also increased an individual's risk for chronic pain, and the suicidality and mental health disorders that accompany this debilitating condition [[4], [5], [6], [7], [8]]. Two adverse perinatal experiences capable of increasing an individual's risk for poor health later in life, are *in utero* intimate partner violence (IPV) and early life neglect [9]. IPV is the most common form of violence inflicted against women, affecting approximately 1/3 of women globally [10,11]. Despite high IPV incidence, little is known about the specific long-term health consequences, and even less about how IPV during pregnancy affects the developing fetus [12,13]. Alternatively, the negative long-term effects associated with early life neglect were unmistakably demonstrated in the studies examining children institutionalized in Romanian orphanages [14]. While the Romanian orphanages are an extreme example of early life neglect, numerous subsequent studies have demonstrated a relationship between less severe physical and emotional neglect early in life and poor health outcomes in adulthood [2,15,16].

Of significance, adolescence represents a critical window for both social development and the emergence of complex health conditions, such as chronic pain [[17], [18], [19]]. The period of heightened plasticity in adolescence likely results from changes in brain structure and function, fluctuations in sex hormones, and age-dependent changes in neuronal maturation [[19], [20], [21]]. Research from neuroimaging studies suggests that the journey of brain maturation is often more important than the destination; meaning that a history of trauma has the potential to disrupt the complex interaction of psychosocial, biological, and environmental factors that contribute to adolescent brain development, possibly conferring increased vulnerability to pathology [19]. Fetal programming and epigenetic mechanisms likely facilitate the emergence of social competencies and/or incompetencies, as well as mental health conditions in adolescence [22]. For example, when ACEs lead to changes in epigenetic processes that modulate the hypothalamic-pituitary-adrenal (HPA) axis, they consequently also change the way an individual responds to a painful stimuli or stressful social interaction [23].

Research demonstrates that maternal psychopathology and adversity, combined with trauma in the offspring, can lead to a cascade of negative intergenerational consequences [9]. However, new findings suggest that social support and the development of effective coping strategies can positively buffer the parent-child dyad [24,25]. Just as prenatal social stress detrimentally influences offspring outcomes [26,27], maternal social support protects offspring from some of the negative sequalae associated with ACEs [24,28]. Given differences in maternal care dynamics in rodent species, it is not possible to increase maternal social support through the addition of supportive conspecifics during the weaning period. Therefore, an alternative strategy is to capitalize on oxytocin's known ability to modulate pain sensitivity, regulate the stress response, and promote positive prosocial behaviours including maternal-offspring bonding [29,30]. Oxytocin is an endogenous hormone and neuropeptide that can easily be administered as a pharmacologic to increase levels of oxytocin within the brain and systemically [29]. Building upon these findings, our study aimed to determine if administration of oxytocin to nursing dams, could remediate the behavioural and neurological deficits in offspring that occur following exposure to perinatal trauma. Given that ACEs rarely occur in isolation, and along with others, we previously demonstrated that the negative effects of these two experiences are cumulative [31,32], we combined the model of *in utero* exposure to IPV with early life neglect, to generate the perinatal adversity, termed perinatal trauma, used within this study to examine the effects on adolescent offspring.

## Methods

### Animals and experimental procedures

**Fig. 1:**
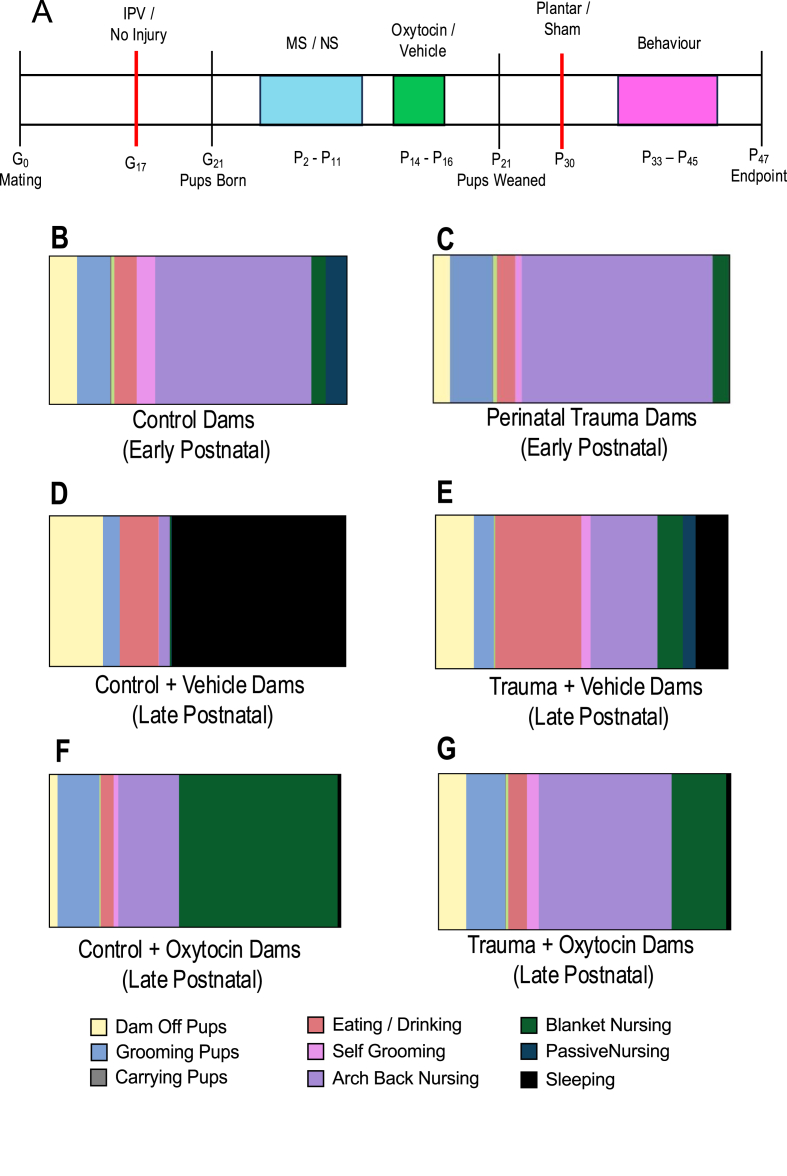
**Timeline and Maternal Behaviour. (A)** Illustrative representation of the experimental timeline. **(B)** Illustrative representation of the percentage of time control dams spent in specific behaviours early in the postnatal period. **(C)** Percentage of time dams exposed to perinatal trauma spent engaged in maternal behaviours early in the postnatal period. **(D)** Illustrative representation of the percentage of time control dams who were administered vehicle spent in specific behaviours late in the postnatal period. **(E)** Illustrative representation of the percentage of time dams exposed to perinatal trauma who were administered vehicle spent in specific behaviours late in the postnatal period. **(F)** Illustrative representation of the percentage of time control dams who were administered oxytocin spent engaged in specific behaviours, measured in the late postnatal period. **(G)** Illustrative representation of the percentage of time dams who were exposed to perinatal trauma and were administered oxytocin spent engaged in specific behaviours, measured in the late postnatal period.

All experiments were approved and carried out in accordance with the Alfred Medical Research and Educational Precinct (AMREP) Animal Ethics Committee (E/8294/2022/M). Adult male and female Sprague Dawley rats, at approximately postnatal day (p) 70, were obtained from the Monash Animal Research Platform (Clayton, Victoria, Australia). They were habituated for 7–10 days to the animal housing facility, which was temperature controlled (21 ​°C) and maintained on a 12:12 light:dark cycle (lights on at 0700). All rats were provided *ad libitum* access to standard laboratory food and water. Following habituation, 12 female rats were mated with 12 distinct male rats, of which 11 females became pregnant. Dams were then randomized such that 6 ​dams were allocated to the perinatal adversity paradigm and 5 ​dams to the sham paradigm. See Fig. 1 for an experimental timeline. Power calculations were completed in G∗Power (version 3.1) using Cohen's (1988) criteria [33], a moderate effect size of 0.45, alpha of 0.05, and a power of 0.80. Given that our final analyses required four-way ANOVAs, this generated an estimated sample size of 120. Therefore, we were adequately powered with our sample size of 125 offspring. Each group contained ∼7–8 animals for behavioural analyses and are identified via individual data points in all graphs.

### Perinatal adversity paradigm

The perinatal adversity paradigm consisted of exposure to both the IPV model during gestation as well as the maternal separation protocol in the neonatal period. The IPV injury, which has been described in detail elsewhere, occurred on gestational day 17 (G17) under isoflurane anaesthesia, and consisted of two parts [32,34]. The first was a mild traumatic brain injury induced via the lateral impact device [35], followed by non-fatal strangulation [31]. For the lateral impact mild traumatic brain injury, the dams were anesthetised with 5 ​% isoflurane at 1 ​l/min O_2_ until non-responsive to a toe-pinch (∼90 ​s) and placed in a prone position in the device. A projectile was propelled towards the temporal lobe of the dam at ∼10.0 ​m/s ± 0.9 using pneumatic pressure. Upon impact the dam was forced into a 180° rotation that generated the rapid acceleration/deceleration forces commonly seen in clinical TBIs. The second part of the injury involved a non-fatal strangulation (i.e., a hypoxic and ischemic insult) where the dams were placed in a supine position prior to a torniquet being placed around their neck. A 250-g weight was added to the torniquet resulting in airway compression and restriction for 90 ​s. Buprenorphine (1 ​mg/kg) and bupivacaine (0.05 ​mg/kg) were administered while the dam was under anaesthesia, between the mild traumatic brain injury and the non-fatal strangulation. Sham dams received the same anaesthetic exposure and analgesic administration but did not undergo impact-induced rotation or experience strangulation. Following the injury induction or sham procedure, dams were placed in the supine position in a warm cage to recover. The time the dam took to right itself following the injury was recorded. All dams who underwent the IPV or sham procedure gave birth on G21. For detailed description of dam characteristics following the IPV injury, please see Ref. [32].

Following birth, dams, and their litters, who were previously randomized to the IPV group, underwent maternal separation (MS). As described elsewhere [36,37], the MS paradigm involved removing the offspring from their dams between p2-p11 for 4 ​h each day. The entire litter was removed from their home cage, weighed, and placed in a separate cage on a warm heating pad. Offspring born to dams in the sham/ no stress (NS) group were removed from their home cages to be weighed but were then immediately returned to their mothers. Given that we have previously characterised the effects of the two IPV components (the lateral impact and the non-fatal strangulation) in combination and as separate entities [34], as well as the effects of the IPV and MS components, in combination and as separate entities [32], for this study, to more accurately reflect human scenarios where ACEs occur in combination, we combined the IPV and MS models to generate a model of perinatal trauma that we could use to examine the efficacy of maternal oxytocin administration.

### Oxytocin administration

When the perinatal adversity paradigm was completed, dams were randomly assigned to receive oxytocin or vehicle treatment, via subcutaneous injection. The oxytocin was administered at a dose of 1 ​mg/kg dissolved in saline and the vehicle was an equal volume of saline. This dosage was selected as it had previously been demonstrated to reduce stress levels and increase nociceptive thresholds in adult female Sprague Dawley rats [38]. Oxytocin or vehicle administration occurred once a day, for 3 days (p14-p16), to dams that were nursing. Although there were no significant effects of oxytocin administration on dam weight, offspring nursing from dams who received oxytocin, exhibited a significant reduction in weight gain over the three days (offspring nursing from dams who received oxytocin gained ∼27 ​% less than offspring whose dam received the placebo, data not shown). At p21, offspring were weaned from their mothers and were randomized into same-sex, same-treatment cages of 3 or 4. At this point there were no significant differences in weight between offspring in the oxytocin group and offspring in the placebo group.

### Maternal behaviour

Dam behaviour was recorded at two time points during the postnatal period: early (between p3-7) and late (between p18-20). When pups were returned to the dam cage, either immediately following the MS paradigm or being weighed, dam behaviour was recorded for 60 ​min. A researcher blinded to the experimental conditions scored each video for the time the dam spent off of the pups, grooming the pups, carrying the pups, nest building, eating and/or drinking, self-grooming, arched back nursing, blanket nursing, passive nursing, and sleeping.

### Plantar incision surgery

At p30 the offspring were randomly assigned to undergo a plantar incision or sham surgery – the surgery induces a mildly painful stimuli that can become chronic in some at risk populations. As described previously [39], the rat was anaesthetised with 5 ​% isoflurane until unresponsive to a toe-pinch, before being transferred to a nose-cone for maintenance anaesthesia with 2 ​% isoflurane. The left hind paw was cleaned using ethanol and chlorhexidine. A scalpel was used to incise the skin and curved forceps were used to isolate the plantaris muscle in the foot. Using the scalpel, the plantaris muscle was incised 3× before the wound was closed with two interrupted sutures. The site was cleaned again with ethanol and chlorhexidine and the rat was placed into a recovery cage. Sham animals were anaesthetised and sutured but did not undergo an incision to the plantaris muscle.

### Behavioural measures

All behavioural testing was completed between 0800 and 1500 ​h by a research associate blinded to the experimental conditions. The behavioural tests were selected as they permitted examination of socio-emotional functioning (the elevated plus maze (EPM), open field, social play, modified forced swim), nociceptive sensitivity (von Frey and hot/cold plate), and cognition (novel context mismatch task (NCM)). The EPM, which was performed on p33, was used to measure anxiety-like behaviour. As described elsewhere [40], the rat was permitted to explore the open and closed arms of the EPM for 5 ​min, while it's movements were captured by overhead tracking software (Topscan Lite Software, Clever Sys Inc., Reston, VA). Open field testing occurred on p34 and examined locomotor behaviour in a 10 ​min session. The rat's movements in the open field were also recorded by the same overhead tracking software. Short-term working memory was then tested with the NCM paradigm [41], which consisted of two habituation days and a testing day (p35-37). The rat's behaviour in the NCM task was captured with a video camera and the time spent investigating the novel object and original object were scored by a research associate blinded to the experimental conditions. Following completion of the NCM task, rats were isolated (singly housed) for 24 ​h, prior to play behaviour testing. Play behaviour testing occurred on p38 under dim red-light; rats were reunited with one of their cage mates of same sex, adversity, and treatment (i.e., in pairs) within a clear Plexiglass box and their behaviour was recorded for 15 ​min. As described by Pellis et al., behaviour was scored frame by frame for the number of attacks, complete rotations, partial rotations, horizontal rotations, evasion, and no-response [42]. Between p40-p44, nociceptive sensitivity was tested using von Frey filaments and the hot/cold plate as indicators of mechanical and thermal sensitivity, respectively [43,44]. Finally, on p45 the modified forced swim task was completed to measure anxiety- and depressive-like behaviors [45]. Rats were individually placed in a tank filled with room temperature water (23 ​°C) and their behaviour was recorded for 7 ​min. A research associate blinded to the experimental conditions documented the amount of time each rat spent immobile.

### Tissue collection and processing

At p47 rats were humanely euthanised via isoflurane anaesthesia and intraperitoneal injection of pentobarbitone prior to rapid decapitation. The somatosensory cortex and hippocampus were dissected, and flash frozen on dry ice. Small skin punches were collected from ear tissue and stored at −20°C for telomere length analyses. Trunk blood was collected in serum separator tubes. Blood was allowed to clot for 30 ​min at room temperature before being centrifuged at 1500*g* at 4 ​°C for 20 ​min. Serum was then aliquoted and stored at −80 ​°C. Half of the serum samples were processed to investigate levels of oxytocin (Enzo Life Sciences, USA) and the other half were processed for CGRP (Elabscience, USA) via ELISAs.

DNA was extracted from ear notch skin samples with the DNeasy Blood and Tissue Kit (Qiagen), according to the manufacturer's protocol. The concentration and purity of samples was measured using the QIAxpert (Qiagen) and samples were subsequently diluted to 30 ​ng/μl. For telomere length, all qRT-PCR reactions were run in duplicate using 1 X SYBR Green FastMix ROX, melting temperatures of 56 ​°C for *Tel* and 54.8 ​°C for *36B4*, on the QuantStudio 7 system (Qiagen) and as previously described [46,47].

Similarly, RNA was extracted and purified from ∼20 ​mg of somatosensory cortex and hippocampus tissue according to the manufacturers protocol using the RNeasy Mini Kit (Qiagen, Hilden, Germany). Quality and concentration of RNA was measured using the QIAxpert (Qiagen). Two micrograms of purified RNA were reverse transcribed to complementary DNA (cDNA) using qScript™ XLT cDNA SuperMix (Quantabio) and used for downstream qRT-PCR. All primers were obtained from IDT. Two housekeeping genes (*Ywhaz* and *Cyca*) were used for normalization in the 2^−ΔΔCt^ method [48]. All samples were run in duplicate on a 384-well plate with a melting temperature of 60 ​°C. Each well contained 20 ​ng of cDNA, 1 X SYBR Green FastMix ROX, and 0.5 ​μM of forward and reverse primers and was run on the QuantStudio 7 system (Qiagen). See Table 1 for all primer sequences and cycling parameters.

**Table 1:** Primer sequences and cycling parameters for qRT-PCR.

Gene symbol	Gene name	Primer sequence	Cycling parameters
*CYCA*	Cyclophilin A	(+) agcactggggagaaaggatt	1 cycle 95 ​°C 20 ​s 40 cycles 95 ​°C 1 ​s 40 cycles Tm°C 20 ​s ​+ ​melt curve
		(−) agccactcagtcttggcagt	
*YWHAZ*	Tyrosine 3-monooxygenase	(+) ttgagcagaagacggaaggt	
		(−) gaagcattggggatcaagaa	
*5HT1A*	Serotonin receptor 1A	(+) ccgcacgcttccgaatcc	
		(−) tgtccgttcaggctcttcttg	
*5HT2A*	Serotonin receptor 2A	(+) aacggtccatccacagag	
		(−) aacaggaagaacacgatgc	
*BDNF*	Brain-derived neurotrophic factor	(+) ccataaggacgcggacttgt	
		(−) gaggctccaaaggcacttga	
*CGRP*	Calcitonin gene related peptide	(+) cagtctcagctccaagtcatc	
		(−) ttccaaggttgacctcaaag	
*DNMT3a*	DNA methyltransferase 3a	(+) gggtgctatctctctttgatgg	
		(−) ctggatatgcttctgtgtgacg	
*ER-*α	Oestrogen receptor alpha	(+) aacatgaaatgcaagaatgtcg	
		(−) tgtaaggaatgtgctgaagtgg	
*GR*	Glucocorticoid receptor	(+) agcttcaggatgtcattacggg	
		(−) gagcttcaaggttcattccagc	
*IBA1*	Ionized calcium-binding adapter molecule 1	(+) tacagcagtgatgaggatctgc	
		(−) ttcagctctagatgggtcttgg	
*MECP2*	Methyl-CpG binding protein	(+) cgtccccttgcctgaaggttgga	
		(−) ctttccagcagagcgaccag	
*OXY-R*	Oxytocin receptor	(+) tcctgtgcctcattctgttcc	1 cycle 95 ​°C 20 ​s 35 cycles 95 ​°C 1 ​s 35 cycles Tm°C 20 ​s ​+ ​melt curve
		(−) gtatttgaccagacgacacagc	
*36B4*	Acidic ribosomal phosphoprotein P0	(+)cagcaagtgggaaggtgtaatcc	1 cycle 95 ​°C 3 ​min 30 cycles 95 ​°C 15 ​s 30 cycles Tm°C 1 ​min ​+ ​melt curve
		(−)cccattctatcatcaacgggtacaa	
*Tel*	Telomere	(+)ggtttttgagggtgagggtgagggtgagggtgagggt	1 cycle 95 ​°C 3 ​min 30 cycles 95 ​°C 15 ​s 30 cycles Tm°C 2 ​min ​+ ​melt curve
		(−)tcccgactatccctatccctatccctatccctatcccta	

The genes were selected given their respective roles in regulating aspects of stress, epigenetic, social, pain, and plasticity processes. We assessed the glucocorticoid receptor (*GR*) due to its role in modulating the stress response, functioning as the receptor to the stress hormone, corticosterone [49]. We also examined the epigenetic regulators DNA methyltransferase 3 alpha (*DNMT3a*) and methyl-CpG-binding protein 2 (*MECP2*), which play significant roles in DNA methylation, development of the central nervous system (CNS), and neuronal plasticity [50,51]. The oxytocin receptor (*OXY-R*) was chosen to evaluate the brain response to peripherally administered oxytocin and correlated behavioural social outcomes [52]. Further associated with social regulation and mood is the serotonin 1A receptor (*5HT1a*) and the serotonin 2a receptor (*5HT2a*), which are often targets for therapeutics [53]. Allograft inflammatory factor 1 (*IBA1*) was assessed as a marker of microglia, which has been correlated with injury and pain [54]. Additionally in regard to pain, we examined calcitonin gene-related peptide (*CGRP*) and the estrogen receptor alpha (*ER-a*) due to their involvement in the transmission and modulation of pain [55,56]. Finally, *ER-a*, as well as brain derived neurotrophic factor (*BDNF*)*,* were also chosen for their roles in CNS development, neuronal plasticity, and behavioural change [[57], [58], [59]].

### Statistical analyses and data availability

For early dam behavioural data, Mann-Whitney tests were run to compare outcomes between dams exposed to Adversity (IPV ​+ ​MS) and control dams (no IPV ​+ ​no MS). For late dam behavioural outcomes, two-way ANOVAs, with Adversity (IPV ​+ ​MS; no IPV ​+ ​NS) and Maternal Oxytocin Administration (Oxytocin; Vehicle) as factors were run. For all offspring behavioural and molecular outcomes, four-way ANOVAs with Sex (Male; Female), Adversity (IPV ​+ ​MS; no IPV ​+ ​NS), Maternal Oxytocin Administration (Oxytocin; Vehicle), and Surgery (Plantar Incision; Sham), as factors were run. All analyses were conducted using SPSS 28.0 for MAC. When appropriate, post-hoc Bonferroni pairwise comparisons were run to examine significant interactions. All figures are displayed as means ​± ​standard error and statistical significance was considered *p* ​< ​0.05. All raw data will be deposited to the open source framework (OSF) repository at https://osf.io/wem5n/?view_only=f00c9515ecfd4999a30dfe3f4f775724.

## Results

### Maternal behaviour

Mann-Whitney U tests were performed to compare maternal behaviours towards their pups for dams exposed to adversity in comparison to dams from the control group. At the early timepoint, exposure to this model of IPV and MS did not affect maternal care/behaviours toward the offspring. There were no significant differences in time the dam spent off of the pups (z ​= ​0.044, *p ​=* ​0.0965), grooming the pups (z ​= ​−1.369, *p ​=* ​0.171), carrying the pups (z ​= ​−0.142, *p ​=* ​0.887), nest building (z ​= ​−0.591, *p ​=* ​0.554), eating and/or drinking (z ​= ​0.139, *p ​=* ​0.890), self-grooming (z ​= ​0.310, *p ​=* ​0.757), arched back nursing (z ​= ​−1.104, *p ​=* ​0.269), blanket nursing (z ​= ​−0.832, *p ​=* ​0.406), passive nursing (z ​= ​0.612, *p ​=* ​0.541), and sleeping (no dams slept). See Fig. 1B and C.

The two-way ANOVAs for maternal behaviour examined at the later timepoint (p18-20) demonstrated that oxytocin treatment reduced the time dams spent off their pups and the time they spent asleep, but increased the time they spent grooming their offspring and engaged in blanket nursing (F_1,10_ ​= ​16.529, *p* ​= ​0.002; F_1,10_ ​= ​29.059, *p* ​< ​0.001; F_1,10_ ​= ​5.733, *p* ​= ​0.044; F_1,10_ ​= ​10.735, *p* ​= ​0.007, respectively). See Fig. 1D–G. Maternal exposure to adversity reduced the amount of time dams spent asleep, but did not affect any other behaviour in isolation (F_1,10_ ​= ​12.668, *p* ​= ​0.005). There were no other significant main effects of adversity or maternal oxytocin administration, *p*'s ​> ​0.05.

### Anxiety-like behaviours

**Fig. 2:**
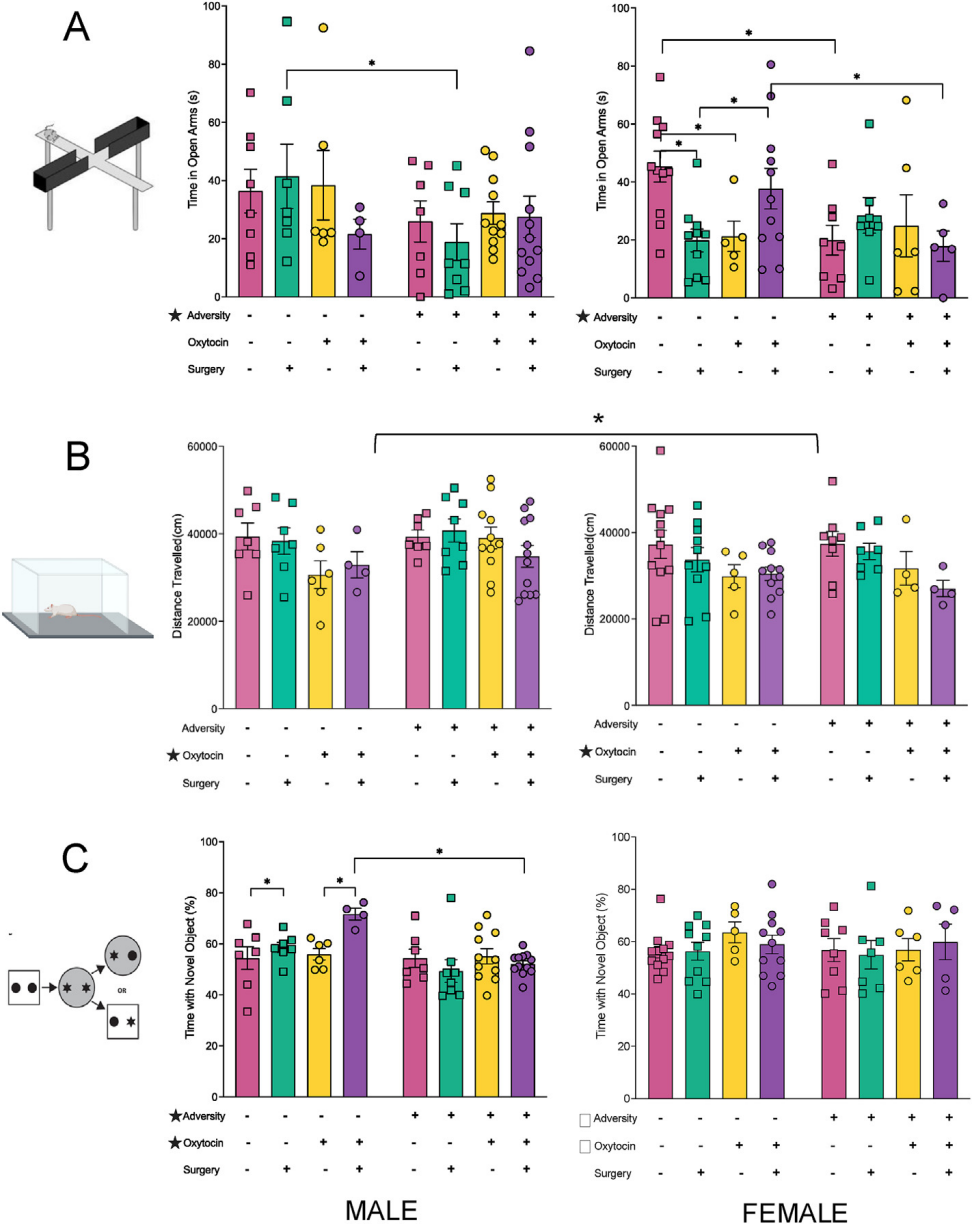
**Socio-emotional and Cognitive Behavioural Results. (A)** Increased anxiety-like behaviour within the elevated plus maze in offspring from the adversity groups that was not remediated with maternal oxytocin administration. **(B)** Male offspring travelled greater distances in the open field than female offspring; locomotor behaviour was reduced in response to maternal oxytocin administration. **(C)** Cognitive performance on the novel context mismatch task was impaired in response to adversity but was improved when oxytocin was administered to the dams. Graphs represent mean ​± ​SEM, and ∗ ​= ​*p* ​< ​.05.

To examine the effects of adversity, surgery, and oxytocin on anxiety-like behaviours we used the EPM and open field tests. Anxiety-like behaviours were increased in rats with early adversity compared to those without as indicated by less time spent in the open arms of the EPM (F_1,124_ ​= ​5.293, *p* ​= ​0.024). See Fig. 2B. The results from the four-way ANOVA for time spent in the open arms of the EPM exhibited a 4-way interaction (F_1,124_ ​= ​7.935, *p* ​= ​0.006). This effect was driven by the plantar incision, maternal vehicle, and no adversity male and female rats (*p* ​= ​0.029). The males who experienced maternal vehicle administration and the plantar surgery who were born to control dams spent more time in the open arms compared to those in the adversity group (*p* ​= ​0.030). Female rats also exhibited alterations in anxiety, whereby those in the adversity group who received the maternal vehicle administration and the sham surgery, spent less time in the open arms compared to those in the no adversity group (*p* ​= ​0.007). Female rats within the no adversity, plantar surgery, and maternal vehicle administration group spent less time in the open arms than those who received maternal oxytocin therapy (*p* ​= ​0.042). Interestingly, female rats in the no adversity, sham surgery, and maternal vehicle administration group also spent more time in the open arms than those who received maternal oxytocin therapy (*p* ​= ​0.027). There was also a trend in female rats for increased anxiety in the offspring who experienced maternal oxytocin administration, a plantar surgery, and adversity, compared to the females with no adversity (*p* ​= ​0.067). On the EPM, female rats within the no adversity, maternal vehicle, and plantar surgery group spent less time in the open arms compared to those who experienced a sham surgery (*p* ​= ​0.004). See Fig. 1B. Finally, in the open field males travelled significantly further than females, and offspring who received maternal oxytocin travelled further than those who received the vehicle (F_1_,_120_ ​= ​6.543, *p* ​= ​0.012, F_1_,_120_ ​= ​12.920, *p* ​< ​0.001, respectively). See Fig. 2C.

### Cognition

We used the novel context mismatch task to assess short term memory. Short-term working memory performance was reduced in offspring from the adversity group (F_1_,_124_ ​= ​5.404, *p* ​= ​0.022), as indicated by less time spent investigating the novel object. Rats who received maternal oxytocin also exhibited increased memory performance (F_1_,_124_ ​= ​4.699, *p* ​= ​0.032). In addition, there was a 3-way interaction between adversity, surgery, and sex (F_1_,_124_ ​= ​4.684, *p* ​= ​0.033), that was driven by the male offspring, in which the males from the plantar surgery and adversity group displayed worse memory performance when compared to the males with no adversity (*p* ​< ​0.001). Further, males born to control mothers who received a plantar surgery had better memory than those with a sham surgery (*p* ​= ​0.027). See Fig. 2D.

### Nociception

**Fig. 3:**
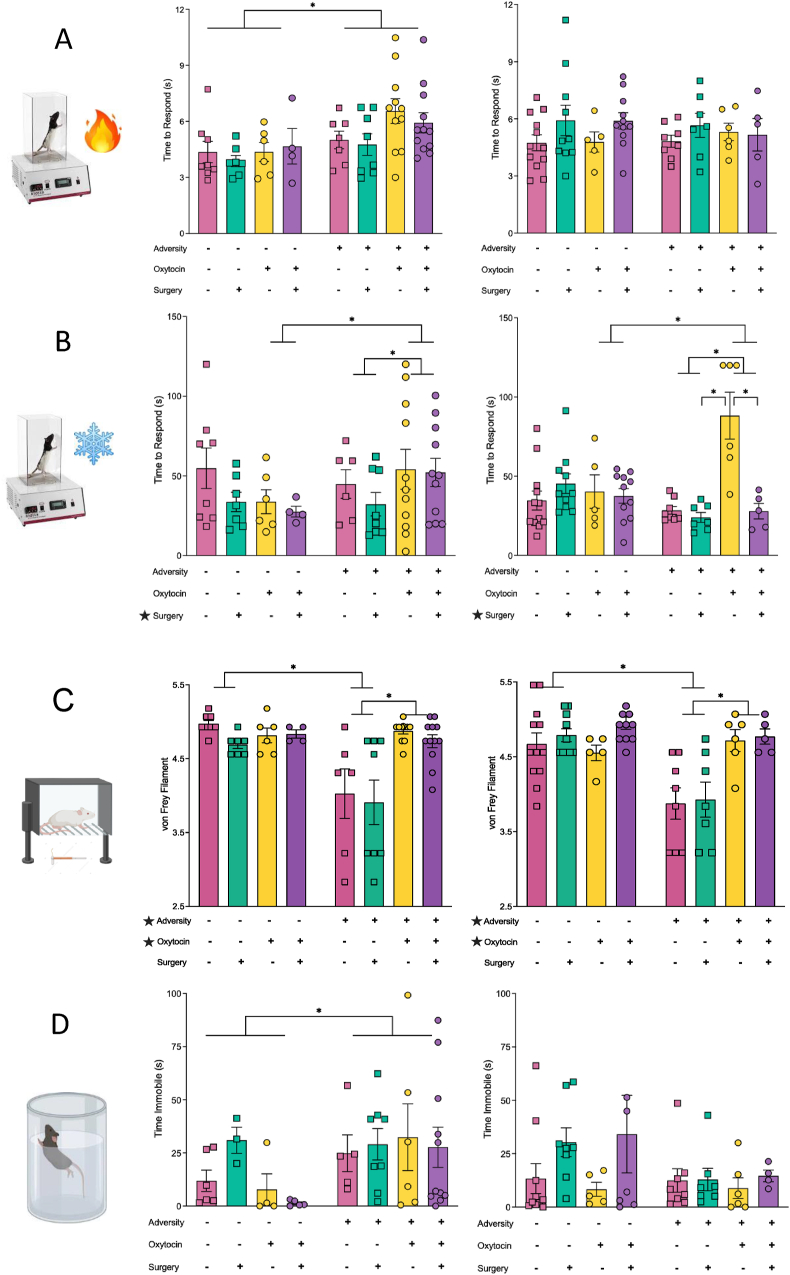
**Nociceptive and Depressive-like Behavioural Results. (A)** Male offspring from the adversity group exhibited a reduction in thermal nociceptive sensitivity on the hot plate. **(B)** Male offspring from the adversity group also displayed reduced nociceptive sensitivity on the cold plate. Conversely, with the exception of the Adversity ​+ ​Oxytocin ​+ ​Sham Surgery group, female offspring from the adversity group exhibited an increase in thermal nociceptive sensitivity on the cold plate. **(C)** Mechanical nociceptive sensitivity as measured with von Frey, was increased in response to adversity, but fixed in response to maternal oxytocin administration, for both male and female offspring. **(D)** Finally, depressive-like behaviours were increased in male offspring exposed to early life adversity as measured with the modified forced swim task. Graphs represent mean ​± ​SEM, and ∗ ​= ​*p* ​< ​.05.

To examine changes in adolescent thermal nociception we used the hot/cold plate. Although there were no main effects identified, the four-way ANOVA revealed a significant interaction between sex and adversity (F_1_,_125_ ​= ​4.422, *p* ​= ​0.038), whereby females born to control dams exhibited reductions in reaction time when compared to males born to control dams (*p* ​= ​0.032). Further, males in the adversity group were less responsive than males in the control group (*p* ​= ​0.008). See Fig. 3A. On the cold plate, there was a significant main effect of plantar surgery, whereby offspring who experienced a plantar surgery exhibited decreased reaction times compared to offspring who experienced a sham surgery (F_1_,_123_ ​= ​6.973, *p* ​= ​0.010). There was also a significant interaction between maternal oxytocin administration and adversity (F_1_,_123_ ​= ​10.656, *p* ​= ​0.001), driven by rats with maternal oxytocin administration and adversity, whereby they exhibited increased reaction times when compared to the no adversity group (*p* ​= ​0.004), and the maternal vehicle plus adversity group (*p* ​< ​0.001). There was a significant three-way interaction between sex, maternal oxytocin, and surgery (F_1_,_123_ ​= ​6.459, *p* ​= ​0.012), whereby females in the maternal oxytocin and a sham surgery group exhibited increased reaction times compared to males in the same group (*p* ​= ​0.038). Conversely, females with maternal vehicle and a sham surgery displayed decreased reaction times when compared to males (*p* ​= ​0.036). See Fig. 3B.

The four-way ANOVA demonstrated that adversity modified mechanical nociception as measured with the von Frey test, whereby offspring who experienced adversity were more sensitive than those with no adversity (F_1_,_124_ ​= ​5.139, *p* ​< ​0.001). Furthermore, maternal oxytocin administration reduced mechanical pain sensitivity (F_1_,_124_ ​= ​5.006, *p* ​< ​0.001). Finally, there was a significant interaction between adversity and maternal oxytocin (F_1_,_124_ ​= ​24.975, *p* ​< ​0.001), whereby offspring who received maternal vehicle in the adversity group had heightened pain sensitivity when compared to offspring who received the vehicle and were not exposed to adversity (*p* ​< ​0.001), as well as when compared to offspring exposed to adversity who received maternal oxytocin (*p* ​< ​0.001). See Fig. 3C.

### Depressive-like behaviour

The modified forced swim task was used to assess changes in anhedonia in adolescence. There were no significant main effects identified in the four-way ANOVA, however there was a significant sex by adversity interaction (F_1_,_100_ ​= ​6.009, *p* ​= ​0.016). Specifically, males in the adversity group spent significantly more time immobile than females in the adversity group (*p* ​= ​0.015), and more time immobile than males in the no adversity group (*p* ​= ​0.042). See Fig. 3D.

### Social behaviour

**Fig. 4:**
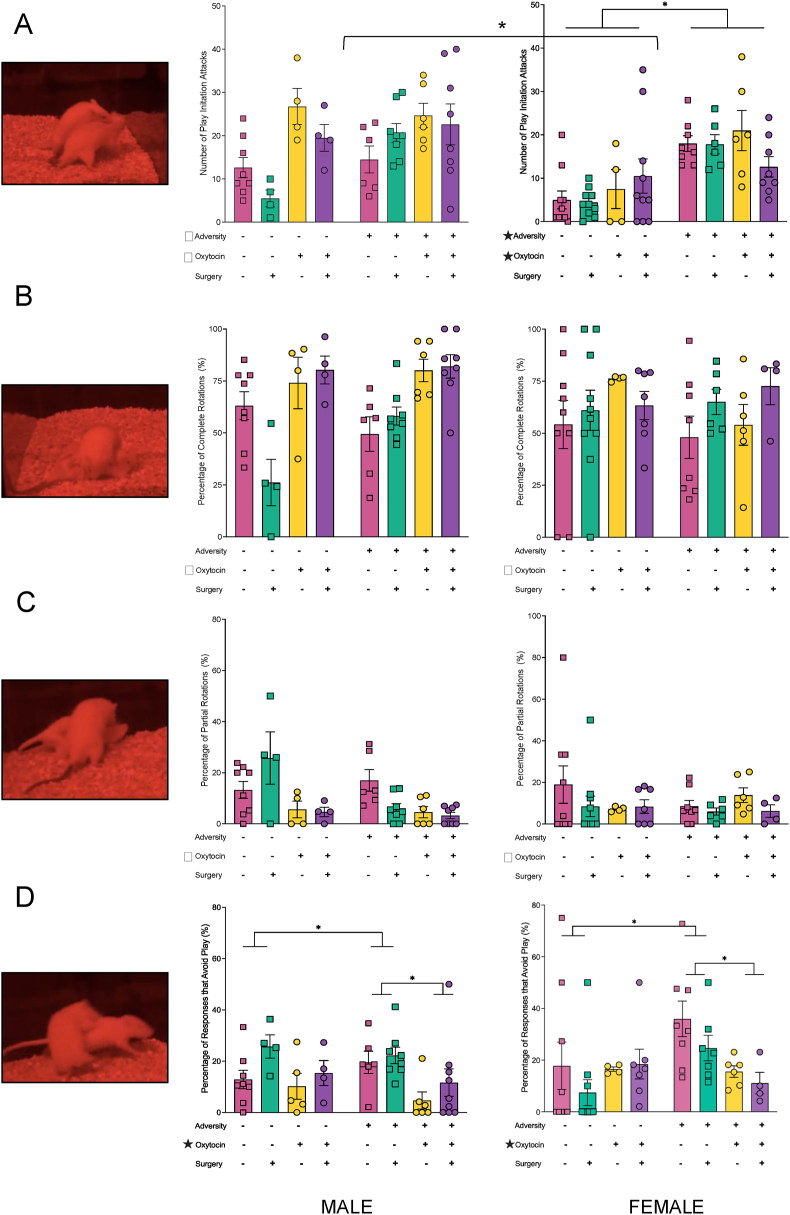
**Illustrative Representation of Play Behaviour Results. (A)** Early life adversity as well as maternal oxytocin administration increased prosocial behaviours and play initiations, with males initiating and engaging in more play than females. **(B)** Maternal oxytocin administration increased prosocial play responses (complete rotations), **(C)** reduced partial rotations, and **(D)** reduced the number of anti-social avoidant play behaviours. Graphs represent mean ​± ​SEM, and ∗ ​= ​*p* ​< ​.05.

Overall, exposure to the adversity paradigm increased prosocial behaviours in adolescent offspring and maternal oxytocin administration reduced the tendency for animals in the adversity group to engage in antisocial behaviours. The four-way ANOVA for play initiations demonstrated a main effect of adversity (F_1, 101_ ​= ​21.253, *p* ​< ​0.001), and a main effect of maternal oxytocin administration (F_1, 101_ ​= ​13.908, *p* ​< ​0.001). Although both factors increased the number of play initiations, the effects were not cumulative. In addition, there was a significant sex by adversity interaction, (F_1, 101_ ​= ​5.024, *p* ​= ​0.028), whereby the effect of adversity was more pronounced in female offspring (*p* ​< ​0.001), see Fig. 4A. Oxytocin administration also increased the positive pro-social responses to play initiation as demonstrated by an increase in the percentage of complete rotations (Fig. 4B). The four-way ANOVA demonstrated a main effect of maternal oxytocin administration, (F_1, 101_ ​= ​12.994, *p* ​< ​0.001). Lastly, maternal oxytocin administration reduced the number of partial rotations in all rats in response to play initiation (F_1, 101_ ​= ​4.040, *p* ​= ​0.048; Fig. 4C), while also reducing the propensity for antisocial responses to play initiation in animals exposed to early life adversity, see Fig. 4D. The four-way ANOVA demonstrated a main effect of oxytocin administration, (F_1, 99_ ​= ​4.899 *p* ​= ​0.030); and a significant adversity by oxytocin administration interaction (F_1, 99_ ​= ​4.086, *p* ​= ​0.045).

### Systemic markers of adversity

**Fig. 5:**
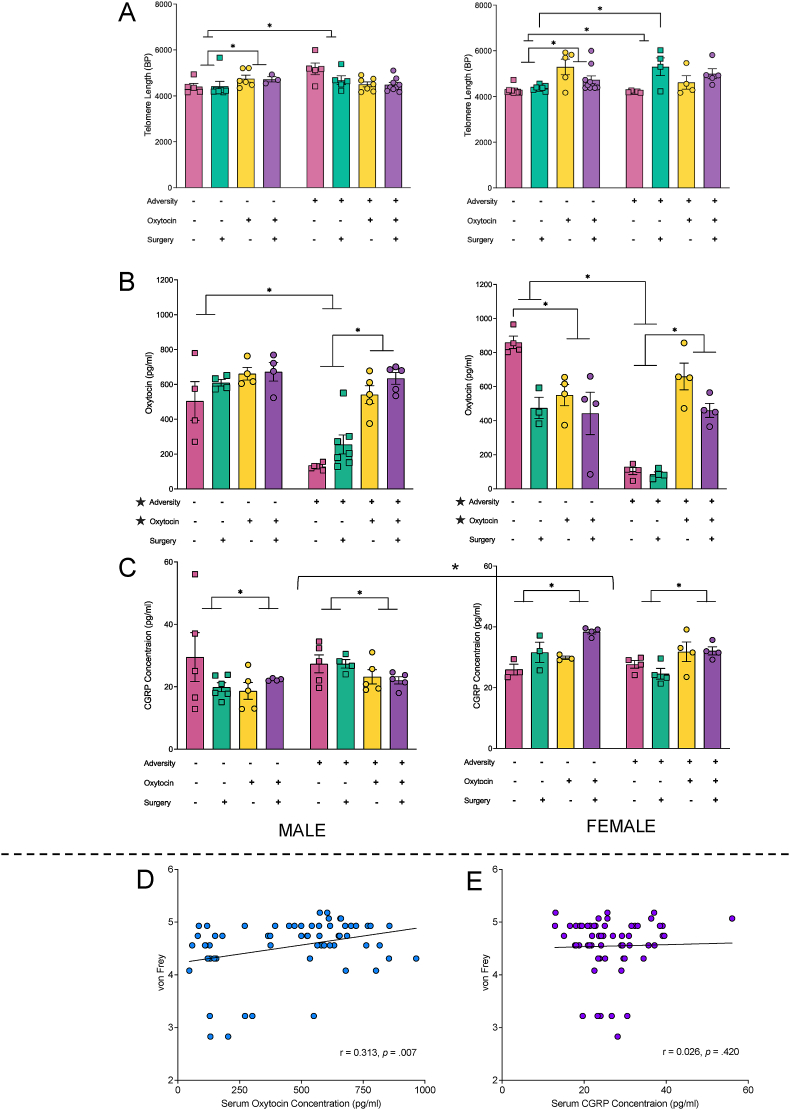
**Systemic markers of adversity. (A)** Adversity increased telomere length in all groups except females ​+ ​adversity ​+ ​vehicle ​+ ​sham surgery. In offspring born to control dams, maternal oxytocin administration increased telomere length. **(B)** Serum oxytocin levels were significantly reduced in offspring exposed to adversity, with the loss of oxytocin being reversed in the maternal oxytocin administration group. **(C)** Female offspring had higher CGRP levels than males; maternal oxytocin administration decreased serum CGRP levels in male offspring but increased serum levels in female offspring. **(D)** A positive correlation was identified between serum oxytocin and nociceptive sensitivity measured with von Frey. **(E)** CGRP levels did not correlate with nociceptive sensitivity. Graphs represent mean ​± ​SEM, and ∗ ​= ​*p* ​< ​.05.

Telomere length was examined in adolescent offspring as a measure of cellular aging and resilience. Although there were no significant main effects, there was a significant interaction between adversity and oxytocin administration (F_1_,_96_ ​= ​14.057, *p* ​< ​0.001). The significant interaction was driven by animals in the maternal vehicle administration, where those in the no adversity group displayed shortener telomere lengths than those in the adversity group (*p* ​< ​0.001), as well as shorter telomeres than offspring in the maternal oxytocin administration group (*p* ​< ​0.001). Furthermore, there was a significant interaction between sex, maternal oxytocin administration, and surgery (F_1_,_96_ ​= ​5.913, *p* ​= ​0.017), whereby males who were administered maternal vehicle and experienced a sham surgery had longer telomere lengths than females in this group (*p* ​= ​0.003). See Fig. 5A.

The serum concentration of oxytocin and CGRP was examined in the offspring using ELISAs. With respect to oxytocin, the four-way ANOVA revealed a main effect of adversity, whereby adversity decreased serum oxytocin concentrations (F_1_,_61_ ​= ​50.151, *p* ​< ​0.001), and a main effect of maternal oxytocin administration, with these groups displaying an increase in oxytocin concentration compared to the vehicle groups (F_1_,_61_ ​= ​26.983, *p* ​< ​0.001). There was also a significant sex by surgery interaction (F_1_,_61_ ​= ​10.589, *p* ​= ​0.002), with post-hoc tests indicating females with the plantar surgery had decreased serum oxytocin compared to females in the sham surgery group (*p* ​= ​0.011). Males displayed the opposite trend, with surgery increasing oxytocin (*p* ​= ​0.062). In offspring that had an adolescent plantar surgery, males had higher serum oxytocin than females (*p* ​= ​0.009), while the sham surgery rats trended to the reverse, with females having higher serum oxytocin (*p* ​= ​0.076). There was also a three-way interaction between sex, maternal oxytocin, and adversity (F_1_,_61_ ​= ​8.277, *p* ​= ​0.006). Female rats in the no adversity plus maternal oxytocin administration group had lowered levels of serum oxytocin than their male counterparts (*p* ​= ​0.021), and, while not reaching significance, this same trend occurred for offspring that received the vehicle treatment (*p* ​= ​0.052). Both male and female offspring in the maternal vehicle administration and adversity group had reduced serum oxytocin compared to the respective no adversity offspring (*p*'s ​< ​0.001). Male rats in the adversity and maternal oxytocin group exhibited increased serum oxytocin compared to males with adversity but maternal vehicle administration (*p* ​< ​0.001). Similarly, female adolescents with adversity had increased serum oxytocin levels when they received maternal oxytocin compared to the group who received the vehicle treatment (*p* ​< ​0.001), while the opposite was true in the females who experienced no adversity (*p* ​= ​0.011). See Fig. 5B.

The four-way ANOVA for serum CGRP levels only identified a significant sex by oxytocin interaction (F_1_,_61_ ​= ​8.054, *p* ​= ​0.007), there were no significant main effects. The interaction was driven by female offspring who experienced maternal oxytocin administration, whereby they exhibited increased serum CGRP when compared to their male counterparts (*p* ​< ​0.001). In addition, female offspring in the maternal oxytocin group had increased serum CGRP when compared to the maternal vehicle group (*p* ​= ​0.048). See Fig. 5C.

We identified a significant positive correlation between serum oxytocin levels and mechanical nociception as measured with the von Frey task (*r* ​= ​0.313, *p* ​= ​0.007), whereby increased serum oxytocin was associated with increased filament size (Fig. 4D). We failed to identify a significant correlation between serum CGRP and mechanical pain sensitivity as measured with von Frey filaments (*r ​=* ​0.026, *p* ​= ​0.420). See Fig. 5E.

### Gene expression results

**Fig. 6:**
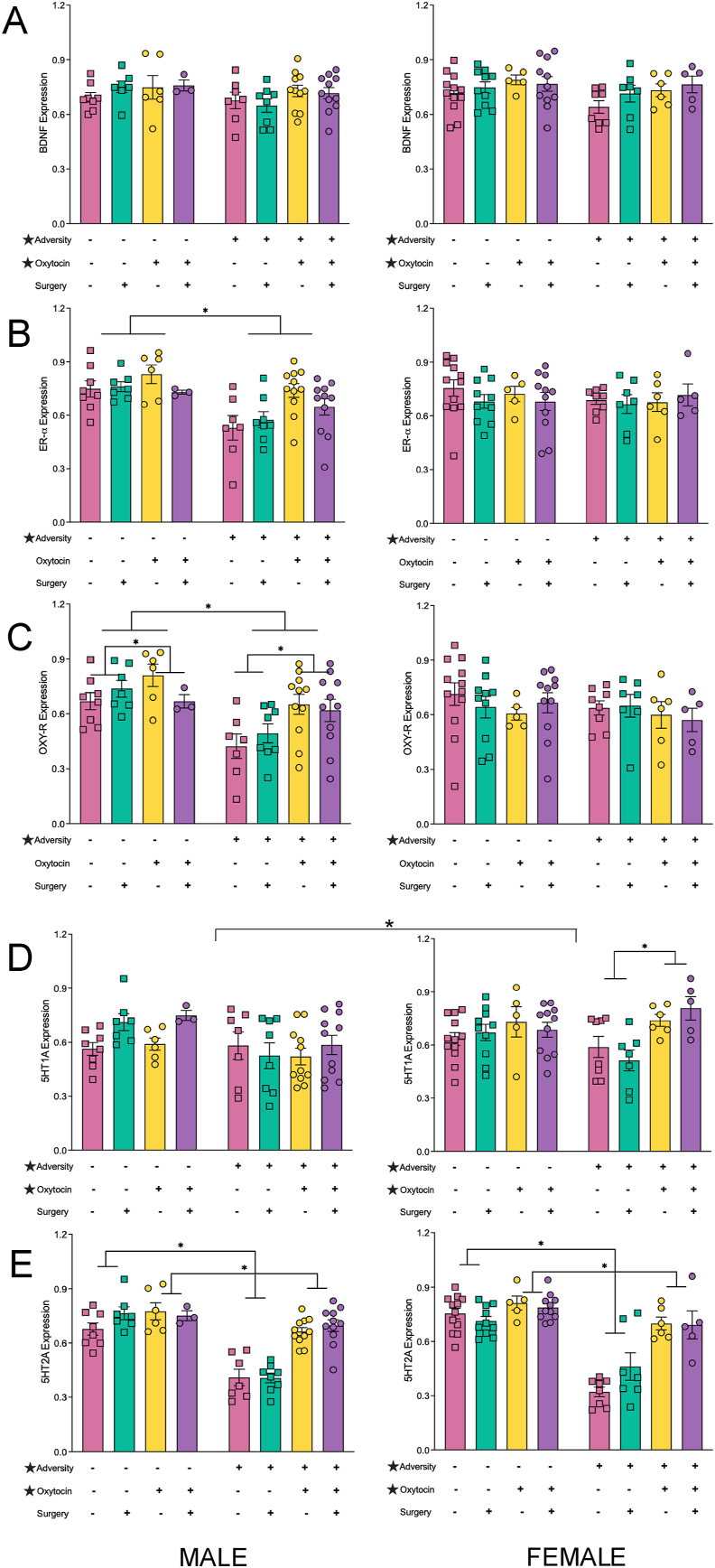
Expression Changes in the Hippocampus for Genes Involved in Neuroplasticity **(A and B)** and Social Behaviours **(C–E)**. Graphs represent mean ​± ​SEM, and ∗ ​= ​*p* ​< ​.05.

**Fig. 7:**
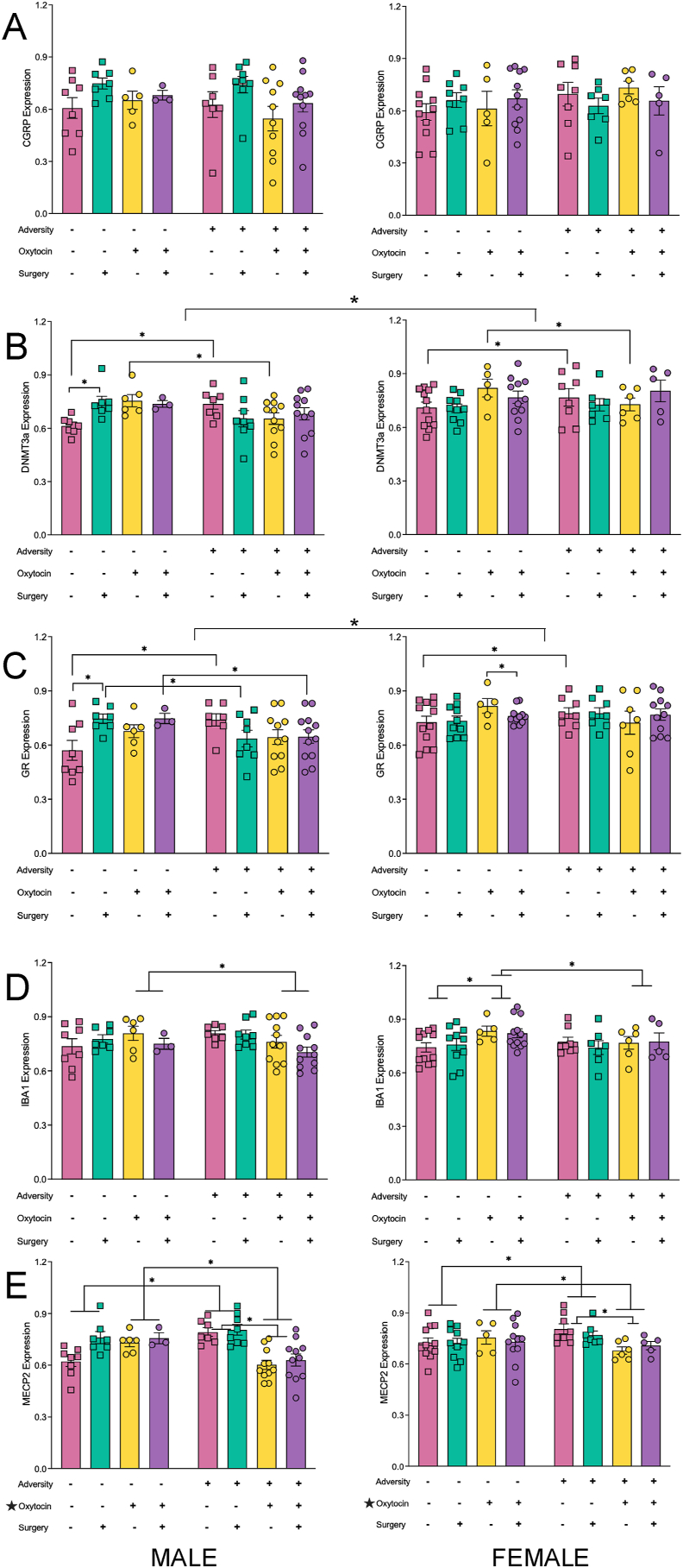
Expression Changes in the Hippocampus for Genes Involved in Pain Sensitivity **(A and D)**, Epigenetic Regulation **(B and E)** and the Stress Response **(C)**. Graphs represent mean ​± ​SEM, and ∗ ​= ​*p* ​< ​.05.

All statistical results for the four-way ANOVAs examining changes in gene expression within the hippocampus and sensory cortex can be found in Table 2, Table 3, respectively. In addition, hippocampus results can be found in graphical representation in Fig. 6, Fig. 7; sensory cortex results in Supplementary Figs. 1 and 2. In brief, exposure to early life adversity had a greater effect on gene expression within the hippocampus as opposed to the sensory cortex. Adversity significantly reduced expression of *BDNF, OXY-R, 5HT1A*, and *5HT2A* in the hippocampus of both males and females, while reducing expression of *ER-α* in the male hippocampus and *IBA1* in the female sensory cortex. Conversely, adversity increased expression of *CGRP* in the sensory cortex of both males and females. When changes in gene expression associated with adversity exhibited sex differences, the effects were more pronounced in males (i.e., Hippocampus – *OXY-R, GR*; Sensory – *BDNF, CGRP, DNMT3a, MECP2, 5HT1A, 5HT2A*).

**Table 2:** Summary of statistical results from the four-way ANOVAs for changes in gene expression within the hippocampus.

Gene	Effect Sex F (p)	Effect Adversity F (p)	Effect Oxytocin Treatment F (p)	Effect Plantar Surgery F (p)	Significant Interactions F (p)
*BDNF*	0.875 (.352)	4.715 (.032)	6.589 (.012)	0.756 (.386)	None
*CGRP*	0.056 (.813)	0.000 (.984)	0.314 (.577)	2.681 (.105)	None
*DNMT3a*	8.322 (.005)	0.437 (.510)	3.377 (.069)	0.050 (.823)	ADV x OXY ​= ​4.255 (.042); ADV x OXY x PLT ​= ​7.310 (.008)
*ER-α*	0.018 (.894)	10.555 (.002)	2.737 (.101)	1.309 (.255)	SEX x ADV ​= ​5.598 (.020)
*IBA1*	0.250 (.618)	0.531 (.468)	0.401 (.528)	0.476 (.492)	SEX x OXY ​= ​4.335 (.040); ADV x OXY ​= ​5.458 (0.021)
*MECP2a*	2.049 (.155)	0.008 (.929)	9.060 (.003)	1.558 (.215)	ADV x OXY ​= ​26.125 (<.001)
*OXY-R*	0.002 (.961)	11.402 (.001)	0.777 (.380)	0.057 (.881)	SEX x ADV ​= ​4.216 (.042); SEX x OXY ​= ​5.973 (.016)
*GR*	14.726 (<.001)	0.096 (.757)	1.363 (.246)	0.510 (.477)	SEX x ADV x PLT ​= ​5.072 (.026); ADV x OXY x PLT ​= ​5.634 (.019)
*5HT1A*	5.656 (.019)	4.265 (.041)	7.238 (.008)	1.724 (.192)	SEX x OXY ​= ​4.594 (.034)
*5HT2A*	0.374 (.542)	113.51 (<.001)	74.82 (<.001)	1.005 (.318)	ADV x OXY ​= ​38.87 (<.001)

**Table 3:** Summary of statistical results from the four-way ANOVAs for changes in gene expression within the sensory cortex.

Gene	Effect Sex F (p)	Effect Adversity F (p)	Effect Oxytocin Treatment F (p)	Effect Plantar Surgery F (p)	Significant Interactions F (p)
*BDNF*	0.098 (.755)	2.587 (.111)	4.556 (.035)	2.743 (.101)	SEX x ADV ​= ​6.966 (.010); SEX x ADV x PLT ​= ​6.254 (.014)
*CGRP*	0.102 (.750)	4.087 (.046)	0.025 (.874)	1.144 (.287)	SEX x ADV x PLT ​= ​4.935 (.028); 4-way interaction ​= ​5.151 (.025)
*DNMT3a*	14.140 (<.001)	0.007 (.935)	0.494 (.484)	0.088 (.768)	SEX x ADV x OXY ​= ​18.874 (<.001)
*ER-α*	1.572 (.213)	0.033 (.587)	7.690 (.007)	4.726 (.032)	SEX x ADV x OXY ​= ​9.503 (.003)
*IBA1*	0.006 (.937)	5.987 (.016)	0.001 (.976)	1.815 (.181)	SEX x PLT ​= ​4.567 (.035); OXY x PLT ​= ​4.475 (.037)
*MECP2a*	1.998 (.160)	0.554 (.458)	5.583 (.020)	0.896 (.346)	SEX x ADV ​= ​8.725 (.004); ADV x OXY ​= ​6.240 (.014); OXY x PLT ​= ​10.755 (.001); SEX x ADV x OXY ​= ​17.88 (<.001)
*OXY-R*	6.163 (.015)	1.061 (.305)	7.574 (.007)	0.600 (.440)	SEX x ADV ​= ​4.277 (.041); SEX x ADV x OXY ​= ​3.977 (.049); SEX x OXY x PLT ​= ​4.375 (.039); ADV x OXY x PLT ​= ​5.024 (.027)
*GR*	0.132 (.717)	0.005 (.942)	1.595 (.209)	0.338 (.562)	SEX x ADV x PLT ​= ​4.678 (.033); SEX x OXY x PLT ​= ​4.314 (.040)
*5HT1A*	0.009 (.925)	2.016 (.159)	0.605 (.438)	0.000 (.997)	ADV x OXY ​= ​30.06 (<.001); SEX x ADV x PLT ​= ​5.682 (.019)
*5HT2A*	1.954 (.165)	0.009 (.926)	1.387 (.241)	0.880 (.350)	SEX x OXY x PLT ​= ​8.054 (.005); 4-way interaction ​= ​4.765 (.031)

We identified a main effect of sex in 4 genes. Within the hippocampus, females exhibited higher expression levels of *5HT1A, DNMT3a* and *GR*. In the sensory cortex, males exhibited higher expression levels of *OXY-R*. The only change in gene expression where we identified a main effect related to the plantar incision surgery was *ER-α*, which was reduced in the sensory cortex of both males and females.

Maternal oxytocin administration normalized changes in gene expression driven by adversity or the plantar incision surgery for both males and females in the sensory cortex expression of *5HT1A,* and the hippocampal expression of *5HT2A* and *MECP2*. Maternal oxytocin administration mitigated negative effects for males only in the expression of *DNMT3a* and *MECP2* in the hippocampus as well as *CGRP* and *OXY-R* in the sensory cortex. Conversely, maternal oxytocin administration attenuated female deficits in *5HT1A* hippocampal expression.

## Discussion

There is robust evidence linking early life adversity to negative long-term health outcomes. Therefore, our next challenge becomes breaking the intergenerational cycle of trauma transmission. For example, given that early exposure to domestic violence is the only ACE directly associated with increased risk for IPV perpetration and physical and/or psychological aggression towards an intimate partner in young adults [60], we need mechanisms and novel preventative approaches to improve offspring trajectories and subsequently disrupt the intergenerational transmission of trauma *before* chronic pain and co-occurring psychopathology begin. Research has demonstrated that maternal social support can buffer many of the negative consequences that occur in response to *in utero* trauma and ACEs [24,28]. However, as ACEs and IPV disproportionately affect individuals from high-risk and low socioeconomic status environments [61], and social support is primarily provided by women [62], it is unreasonable to mandate additional caregiver responsibilities to already overburdened mothers. Hence, a treatment option that is easily implemented across a variety of environments, is lost-cost, scalable, and long-lasting, is urgently needed [63]. Therefore, we investigated whether short-term oxytocin administration could remediate some of the functional impairments associated with exposure to perinatal trauma, and subsequently reduce the risk for pathologies such as chronic pain in adolescence. Oxytocin, often referred to as the ‘love drug’, is involved in social behaviour, maternal care, and pair bonding [29]. However, the dark side of oxytocin – its ability to induce antisocial, aggressive, and fear-related behaviours – particularly when an individual is under extreme stress [64], also need to be considered. This is an especially important concept given the circumstances surrounding its administration in the scenario under investigation.

Overall, we found that adversity detrimentally affected most of the behaviours we examined and had a larger negative impact on males. This is consistent with findings from prenatal stress, as well as *in utero* inflammation studies (i.e., the flu), where males are more negatively impacted by inflammation, and are therefore much more vulnerable to a range of neurodevelopmental disorders [65,66]. We found that exposure to adversity impaired cognitive performance and increased depressive-like behaviours in male offspring only; female behaviours in these domains were unaffected. This is similar to a study by Iturra-Mena et al., who found that compared to females, prenatally stressed males exhibited a dampening of HPA axis activity which was associated with an increase in depressive-like behaviours [67]. We also found that adversity increased anxiety-like behaviours in both sexes, with maternal oxytocin administration failing to provide any protection. Although oxytocin is known to have strong anxiolytic effects and many researchers believe that its prosocial benefits result from suppression of socially-mediated anxiety [29,30,68], it appears that the anxiolytic effects of oxytocin were not transmitted from mothers to their offspring, or alternatively that the anxiolytic effects of oxytocin were not long-lasting.

It is important to highlight, that within this study, maternal oxytocin treatment likely moderated numerous processes to influence offspring outcome. First, oxytocin, or its metabolites, were likely transferred directly to offspring via lactation and nursing, as offspring within the oxytocin group experienced significant weight loss throughout the 3 days of maternal oxytocin administration. It is therefore plausible, that offspring within the maternal oxytocin group experienced oxytocin-induced anorexigenic signalling, that often leads to weight loss [69]. In addition, maternal oxytocin administration restored reductions in *OXY-R* expression induced by the adversity paradigm, suggesting that increased oxytocin levels were detected within the offspring brain, which subsequently modified the transcriptome in this group. Lastly, and consistent with previous literature highlighting the pro -social and -bonding capacity of oxytocin [29,70], we found that maternal oxytocin administration increased the time that dams spent with their pups, specifically the time engaged in nursing and grooming behaviours. Changes in maternal care patterns likely had long-lasting effects on offspring outcomes within this study, as they have consistently been linked with alterations to neuronal architecture, modifications to stress reactivity, and changes to the social and cognitive behaviours of their offspring [37,71,72].

Adolescence is another critical window for the optimization of social behaviours; compared to adults or children, adolescents are more social, form more complex and hierarchal social groups, and are more sensitive to social judgement [17,73]. As anticipated, male offspring born to dams in the control group exhibited reductions in prosocial behaviour and increased anti-social behaviour following the plantar incision surgery. Interestingly, the pain-induced impairment in play behaviour was reversed if their mother received oxytocin treatment, suggesting that, just as early life adversity can prime the brain to be more susceptible to painful stimuli later in life [74,75], positive experiences such as increased maternal care, can be neuroprotective and promote resiliency.

Contrary to our expectations, offspring exposed to perinatal trauma exhibited increases in prosocial behaviour and initiated play significantly more often than offspring born to control dams. This was unexpected but could be a compensatory response. It is possible that offspring in the adversity group experienced reductions in positive maternal care and prosocial behaviours such as licking and grooming [76]. In an effort to compensate for these reductions, adolescents may have initiated more play to re-establish the homeostatic balance that underpins dyadic social/play encounters [77]. Alternatively, ACEs have been linked to increased risk for addiction and substance abuse disorders [2,78]. Given that play is a highly rewarding activity and adolescence represents a period of heightened risk for the emergence of addiction-related disorders, it is possible that the adversity paradigm modified reward pathways [78], leading to increased sensitivity to the pleasurable effects of play.

In animals exposed to early adversity, there was an increase in avoidant play responses, consistent with previous research demonstrating that prenatal stress is linked to increased fear responsivity and avoidant behaviours in offspring [79]. Although maternal oxytocin administration had no effect on the amount of prosocial play initiated or play continuation behaviours, it did reduce the propensity for antisocial avoidant play behaviours. The finding that the benefits of maternal oxytocin administration could improve offspring social behaviours was particularly interesting, because while oxytocin is known to enhance social abilities in the individual receiving the pharmacologic [80,81], this is one of the first studies to demonstrate that these benefits can be passed in a stable manner, from mothers to their offspring.

Corroborating previous ACE studies [6,7,75], our adversity paradigm significantly increased mechanical nociceptive sensitivity providing further evidence that early life adversity can increase an individual's susceptibility to allodynia and chronic pain. Of significance, maternal oxytocin administration completely mitigated this hypersensitivity suggesting that offspring resiliency and neuroprotection can be induced through maternal interventions. Interestingly, circulating serum levels of oxytocin were also significantly reduced in offspring exposed to the adversity paradigm, but the three days of maternal oxytocin therapy was able to remediate this deficit. This supports the hypothesis that a critical window for the oxytocin system may exist within the weaning period [82]. Moreover, we identified a positive correlation between circulating oxytocin levels and mechanical sensitivity, which highlights the analgesic effects of oxytocin [83], and suggests that the adversity-induced reductions in oxytocin contributed to the increases in nociceptive sensitivity. Conversely, although circulating CGRP levels are consistently linked to migraine pain [55,84], we found no correlation between mechanical nociceptive sensitivity and serum CGRP.

Environmental or stimulus-based events can drive changes in gene expression which facilitate an individual's capacity for change – in some circumstances leading to adaptation and in other situations, vulnerability [85]. There are critical windows, such as prenatally, early in life, and in adolescence, whereby these circumstances have a greater likelihood of inducing epigenetic change [9,22,86]. Within this study, the adversity paradigm significantly modified the transcriptome within the brain, having a greater effect on gene expression in the hippocampus than the sensory cortex. This molecular vulnerability may be related to the high degree of neuroplasticity that exists across the lifespan in the hippocampus [87]. Moreover, the hippocampus is known to be particularly vulnerable to the effects of stress and intergenerational adversity, as it has a delicate and complex relationship with the HPA-axis, and contains an abundance of high and low affinity glucocorticoid receptors [88,89]. It is possible that the adversity-induced increases in hippocampal *GR* expression we identified, were compensatory to accommodate for increased glucocorticoid release early in life.

In line with the behavioural findings, when adversity interacted with sex, the transcriptomic effects were more pronounced in males. The larger effects in males may have been related to their changes in *OXY-R*. In both brain regions examined in males, the adversity paradigm significantly reduced expression of *OXY-R,* while maternal oxytocin administration was able to restore this deficit. Conversely, adversity had no effect on female *OXY-R* expression in either brain region. *OXY-R* expression in females was most substantially influenced by the plantar incision surgery in adolescence. Given that the activity of endogenous oxytocin is pleiotropic and contributes to the promotion of allostasis [90], the prominent adversity-induced changes in male *OXY-R* levels may be linked to their heightened impairment.

*DNMT3a* and *MECP2* are both involved in modulating neuroplasticity by regulating expression of other genes. Both genes are highly involved in epigenetic processes necessary for maturation of the CNS and for maintaining plasticity within mature neurons [51,91]. Within the hippocampus, adversity increased expression of both *DNMT3a* and *MECP2* but these changes were mitigated by maternal oxytocin administration, suggesting that oxytocin administration reversed the suppression of neuroplasticity that was likely induced through increased *DNMT3a* and *MECP2* activity. In contrast to our findings, previous rodent studies found that low levels of maternal care and MS reduced *DNMT3a* levels within the hippocampus early in life (∼p7 and p17) [92,93] suggesting that early life adversity does not result in static changes to the transcriptome, but rather interacts with the environment to modulate epigenetic regulation of neuroplasticity throughout development. Human studies have found that reduced *MECP2* expression in blood predicted psychopathology and risk for post-traumatic stress disorder in female adults, but not male adults with a history of ACEs [94,95]. Our contradictory findings of adversity related increases in *MECP2* expression, and worse behavioural manifestations in male offspring, may be because we examined gene expression in the brain, rather than systemically, and explored early adolescence, not adulthood. Irrespective of the dynamics, our findings suggest that perinatal adversity is capable of modulating epigenetic regulators of neuroplasticity and that some of the benefits related to oxytocin therapy may result from re-establishment of their homeostatic levels.

Gonadal hormones are known to modulate HPA axis activity through binding of the *ER-α* receptor, whereby activation of *ER-α* inhibits the glucocorticoid negative feedback loop [96]. In males we found that adversity reduced *ER-α* expression in the hippocampus, possibly in a compensatory fashion, to supress overactivity of the HPA-axis. Interestingly this reduction in hippocampal *ER-α* expression was reversed following maternal oxytocin administration, providing further support for the beneficial effects of oxytocin. Within the sensory cortex, *ER-α* plays a more significant role in social recognition, social learning, and social cognition, also contributing to the development and maintenance of sexually dimorphic behaviours [58]. Interestingly, in this brain region, adversity appeared to feminize the male brain (the expression pattern resembles females born to control dams) and masculinize the female brain (the expression pattern of females born in the adversity group resembles males born to control dams). There are previous studies suggesting that prenatal stress can reduce the extent and degree of sexually dimorphic behaviours such as copulation and lordosis [97,98]. Moreover, our social play data supports this hypothesis. Female adolescents generally initiate play less often than males (as demonstrated in Fig. 3A); however, following exposure to early life adversity, the females carried out significantly more play attacks, to the point that they resembled male offspring.

The response of the serotonergic system, at least as examined via changes in *5HT1A* and *5HT2A* expression, exhibited adversity-induced impairments that were attenuated with maternal oxytocin administration, albeit these modifications occurred in a sex and brain region-specific manner. Hippocampal serotonin receptor changes have been linked to behavioural impairments associated with MS in young rats [99] and repeated trauma exposure in adult rats [100]. In addition, extensive evidence suggests that individual variations in *5HT1A* expression and activity modulate individual responses to stress and trauma [101], thereby suggesting that the adversity-induced changes in *5HT1A* expression have the potential to modify how the offspring would respond to stressful or adverse experiences in the future. Many of the human studies demonstrating dysregulation of serotonin receptor expression associated with stress and trauma, are confounded by their inability to determine if the changes in *5HT1A* and *5HT2A* are caused by stress exposure or represent a pre-existing risk factor for vulnerability to trauma-related disorders [101]. Our findings suggest that changes to these genes occur as a result of the trauma-exposure, but importantly, are responsive to pharmacological treatment.

In summary, we demonstrate that exposure to early life adversity (*in utero* IPV combined with neonatal maternal separation) negatively influenced cognitive, emotional, and social functioning in adolescence, with these behavioural deficits being associated with gene expression changes in the brain. For many of the factors analysed, adversity modified the offspring's response to the plantar incision surgery, providing further support that early life experiences can increase an individual's risk for the development of chronic pain. Of critical significance, however, we also showed that maternal oxytocin administration during nursing was capable of mitigating many of the negative consequences associated with adversity, including impaired social behaviour and allodynia. It is important to note however, that, as with all intergenerational studies, there are limitations associated with litter effects and the associated reductions in molecular phenotypes [102]. However, given that across each of the conditions examined in this study, there were no more than 2 pups/condition (1 male:1 female) originating from a single dam, and we used primiparous females mated with genetically distinct males, we are confident that we have minimized litter effects, as much as possible, while adhering to current animal ethics guidelines [103]. Furthermore, our exciting findings suggest that future studies should explore whether the ability of social support to buffer children from the negative effects of ACEs, is also mediated by the oxytocin system in human populations. It would also be important to understand the parameters surrounding the critical window for oxytocin in humans, so that medically approved oxytocin nasal sprays and pharmacotherapies could be applied across the most efficacious time periods. Lastly, future research should focus on understanding if and how the benefits of oxytocin therapy and/or resiliency to negative health outcomes can be transmitted intergenerationally. There has been recent exploration into mechanisms that promote resiliency to pain [104], and although this field is in its infancy, understanding how to promote generational transmission of these circuits and factors would be invaluable.

## Author contributions

SH was responsible for investigation, data curation, validation, formal analysis, writing the original draft, writing review/editing, and visualization.

ZK was responsible for investigation, data curation, writing the original draft, and writing review/editing.

SS was responsible for investigation, data curation, writing the original draft, and writing review/editing.

MSgro was responsible for investigation, data curation, writing the original draft, and writing review/editing.

EV was responsible for investigation, data curation, writing the original draft, and writing review/editing.

CNL was responsible for investigation, data curation, writing the original draft, and writing review/editing.

MSmith was responsible for investigation, data curation, writing the original draft, and writing review/editing.

SRS was involved in study conceptualisation, methodology, supervision, writing the original draft, and writing review/editing.

GRY was involved in study conceptualisation, investigation, data curation, supervision, writing the original draft, and writing review/editing.

MN was involved in study conceptualisation, supervision, writing the original draft, and writing review/editing.

RM was responsible for conceptualisation, methodology, validation, formal analysis, investigation, resources, data curation, writing the original draft, writing review/editing, visualization, supervision, project administration, and funding acquisition.

## Funding source

The authors would like to thank the 10.13039/501100000925National Health and Medical Research Council of Australia for their financial contribution through the Investigator Grant funding scheme – Grant number 1173565.

## Declaration of competing interest

The authors declare the following financial interests/personal relationships which may be considered as potential competing interests: Richelle Mychasiuk reports financial support was provided by National Health and Medical Research Council. If there are other authors, they declare that they have no known competing financial interests or personal relationships that could have appeared to influence the work reported in this paper.
