# Supplementary material for: Maternal oxytocin administration mitigates nociceptive, social, and epigenetic impairments in adolescent offspring exposed to perinatal trauma
Source: Neurotherapeutics. 2025 Apr 22;22(4):e00598. doi: 10.1016/j.neurot.2025.e00598 (PMC12418415; doi:10.1016/j.neurot.2025.e00598)
Supplement: Multimedia component 1 [file mmc1.docx]

**Supplementary Figures**

**Figure 1.** Expression Changes in the Sensory Cortex for Genes Involved in Neuroplasticity (A and B) and Social Behaviours (C-E). Graphs represent mean ± SEM, and * = *p* < .05. Main effects are indicated with
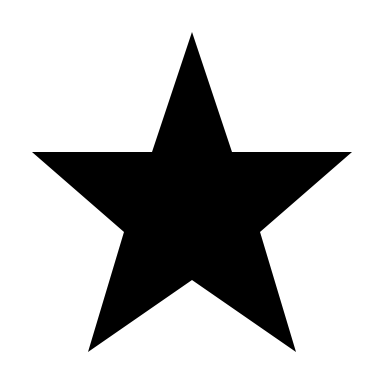
.

**Figure 2.** Expression Changes in the Sensory Cortex for Genes Involved in Pain Sensitivity (A and D), Epigenetic Regulation (B and E) and the Stress Response (C). Graphs represent mean ± SEM, and * = *p* < .05. Main effects are indicated with
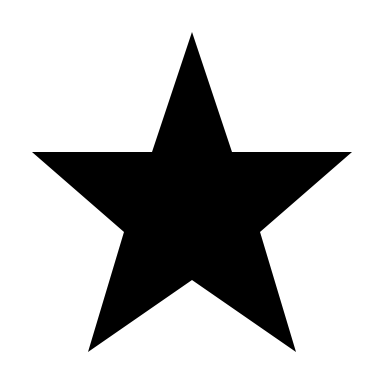
.
